# Supplementary material for: Understanding the Biophysical Interaction of LTX-315 with Tumoral Model Membranes
Source: Int J Mol Sci. 2022 Dec 29;24(1):581. doi: 10.3390/ijms24010581 (PMC9820754; doi:10.3390/ijms24010581)
Supplement: Supplementary file 1 [file ijms-24-00581-s001.zip › ijms-2065477-supplementary.pdf]

Supplementary Files

Figure S1. HPLC-MS chromatogram of the peptide LTX-315.

Sample Name :LTX
Sample ID :U5663DI180-5
Time Processed : 9:18:56
Month-Day-Year Processed :10/24/2018

Pump A : 0.065% trifluoroacetic in 100% water (v/v)
Pump B : 0.05% trifluoroacetic in 100% acetonitrile (v/v)
Total Flow:1 ml/min
Wavelength:220 nm
<<LC Time Program>>

Time	Module	Command	Value
0.01	Pumps	Solvent B Conc.	5
25.00	Pumps	Solvent B Conc.	65
25.01	Pumps	Solvent B Conc.	95
27.00	Pumps	Solvent B Conc.	95
27.01	Pumps	Solvent B Conc.	5
33.00	Pumps	Solvent B Conc.	5
33.01	Controller	Stop	

<<Column Performance>>
<Detector A>
Column : AlltimaTM C18 4.6 x 250 mm
Equipment:ZJ17010507

<Chromatogram>

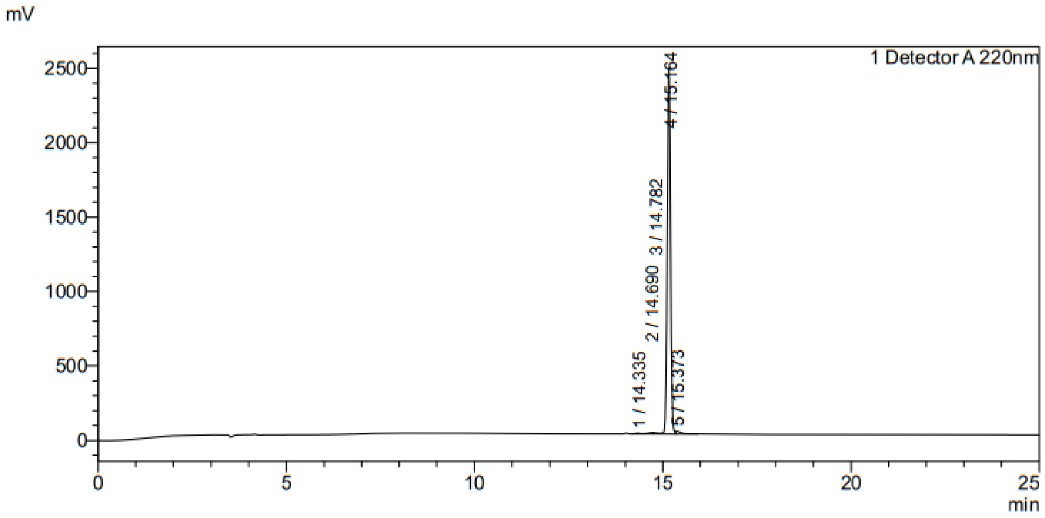

<Peak Table>

Detector A 220nm

Peak#	Ret. Time	Area	Height	Area%
1	14.335	42388	4807	0.270
2	14.690	91336	9444	0.582
3	14.782	61859	9028	0.394
4	15.164	15289226	2462846	97.451
5	15.373	204305	20111	1.302
Total		15689114	2506237	100.000

Table S1. Thermodynamic parameters of main phase transition and pretransition P_β (#) of pure, fully hydrated DPPC multilamellar liposomes and DPPC/Peptide mixtures determined from heating scans collected at a heating rate of 1°C min^{-1}. The accuracy for the main phase transition temperature and enthalpy was ± 0.01°C and ± 0.8 kJ/mol, respectively.

	Heating		
	**T [°C]**	**ΔH [kJ mol^{-1}]**	**ΔS [kJ mol^{-1} K^{-1}]**
**DPPC**	42.20<br>35.84 (P_β)	32.37<br>2.84	0.1026<br>0.0082
+ 1 mol% LTX-315	42.10<br>35.48(#)	29.88<br>2.22	0.0948<br>0.0072
+ 5 mol% LTX-315	42.10<br>35.41	28.99<br>1.98	0.0920<br>0.0064
+ 10 mol% LTX-315	42.17<br>35.48	26.52<br>2.34	0.0841<br>0.0076

Table S2. Thermodynamic parameters of main phase transition and pretransition of pure, fully hydrated DPPE multilamellar liposomes and DPPE/Peptide mixtures determined from heating scans collected at a heating rate of 1°C min^{-1}. The accuracy for the main phase transition temperature and enthalpy was ± 0.01°C and ± 0.8 kJ/mol, respectively.

	Heating		
	**T [°C]**	**ΔH [kJ mol^{-1}]**	**ΔS [kJ mol^{-1} K^{-1}]**
**DPPE**	65.04	35.60	0.1053
+ 1 mol% LTX-315	65.11	32.76	0.0969
+ 5 mol% LTX-315	65.07	32.49	0.0961
+ 10 mol% LTX-315	64.95	34.72	0.1027

Table S3. Thermodynamic parameters of main phase transition and pretransition of pure, fully hydrated DPPS multilamellar liposomes and DPPS/Peptide mixtures determined from heating scans collected at a

heating rate of 1°C min^{-1}. The accuracy for the main phase transition temperature and enthalpy was ± 0.01°C and ± 0.8 kJ/mol, respectively.

	Heating		
	T [°C]	ΔH [kJ mol^{-1}]	ΔS [kJ mol^{-1} K^{-1}]
**DPPS**	54.69	35.48	0.1082
+ 1 mol% LTX-315	54.55	27.84	0.0850
+ 5 mol% LTX-315 *	53.18 and 54.33	38.40	0.1173
+ 10 mol% LTX-315 *	50.70, 53.74 and 54.85	42.92	0.1309

*** 2 or more phases**

Table S4. Thermodynamic parameters of main phase transition and pretransition of pure, fully hydrated SM multilamellar liposomes and SM/Peptide mixtures determined from heating scans collected at a heating rate of 1°C min^{-1}. The accuracy for the main phase transition temperature and enthalpy was ± 0.01°C and ± 0.8 kJ/mol, respectively.

	Heating		
	T [°C]	ΔH [kJ mol^{-1}]	ΔS [kJ mol^{-1} K^{-1}]
**SM**	39.73	42.0	0.1342
+ 1 mol% LTX-315	39.56	35.31	0.1129
+ 5 mol% LTX-315	38.87	39.04	0.1251
+ 10 mol% LTX-315	38.43	42.80	0.1374
